# Computer-assisted three-dimensional quantitation of programmed death-ligand 1 in non-small cell lung cancer using tissue clearing technology

**DOI:** 10.1186/s12967-022-03335-5

**Published:** 2022-03-16

**Authors:** Yen-Yu Lin, Lei-Chi Wang, Yu-Han Hsieh, Yu-Ling Hung, Yung-An Chen, Yu-Chieh Lin, Yen-Yin Lin, Teh-Ying Chou

**Affiliations:** 1 Department of Pathology and Laboratory Medicine, Taipei Veterans General Hospital, No.201, Sec. 2, Shipai Rd., Taipei, 11217 Taiwan; 2 Institute of Clinical Medicine, National Yang Ming Chiao Tung University, Taipei, Taiwan; 3 JelloX Biotech Inc., Hsinchu, Taiwan; 4 Brain Research Center, National Tsing Hua University, Hsinchu, Taiwan

**Keywords:** Non-small cell lung cancer, Immunotherapy, PD-L1, Tissue clearing, 3D imaging, Artificial intelligence

## Abstract

Immune checkpoint blockade therapy has revolutionized non-small cell lung cancer treatment. However, not all patients respond to this therapy. Assessing the tumor expression of immune checkpoint molecules, including programmed death-ligand 1 (PD-L1), is the current standard in predicting treatment response. However, the correlation between PD-L1 expression and anti-PD-1/PD-L1 treatment response is not perfect. This is partly caused by tumor heterogeneity and the common practice of assessing PD-L1 expression based on limited biopsy material. To overcome this problem, we developed a novel method that can make formalin-fixed, paraffin-embedded tissue translucent, allowing three-dimensional (3D) imaging. Our protocol can process tissues up to 150 μm in thickness, allowing anti-PD-L1 staining of the entire tissue and producing high resolution 3D images. Compared to a traditional 4 μm section, our 3D image provides 30 times more coverage of the specimen, assessing PD-L1 expression of approximately 10 times more cells. We further developed a computer-assisted PD-L1 quantitation method to analyze these images, and we found marked variation of PD-L1 expression in 3D. In 5 of 33 needle-biopsy-sized specimens (15.2%), the PD-L1 tumor proportion score (TPS) varied by greater than 10% at different depth levels. In 14 cases (42.4%), the TPS at different depth levels fell into different categories (< 1%, 1–49%, or ≥ 50%), which can potentially influence treatment decisions. Importantly, our technology permits recovery of the processed tissue for subsequent analysis, including histology examination, immunohistochemistry, and mutation analysis. In conclusion, our novel method has the potential to increase the accuracy of tumor PD-L1 expression assessment and enable precise deployment of cancer immunotherapy.

**Supplementary Information:**

The online version contains supplementary material available at 10.1186/s12967-022-03335-5.

## Introduction

The development of immune checkpoint blockade therapy has revolutionized cancer treatment. This modality of treatment, by unleashing the patients’ immune system from cancer suppression, has produced durable responses in multiple types of cancer even in the advanced stage, especially in non-small cell lung cancer [1]. The immune checkpoint programmed death-1 (PD-1)-programmed death-ligand 1 (PD-L1) axis is the major target of current therapies. Blocking antibodies to the axis are effective in treating several cancer types including lung cancer, bladder cancer, head and neck cancer, and melanoma [2]. However, not all patients responded to the treatment. Evidence showed that the variable response can be partially explained/predicted by the tumor expression of PD-L1. In particular, lung cancer patients with more than 50% tumor cells expressing PD-L1 are more likely to respond to the anti-PD-1 antibody, pembrolizumab [3]. Therefore, evaluation of tumor PD-L1 expression by a pathologist has become a standard procedure in selecting patients for therapy. However, the correlation between PD-L1 expression and clinical response is not perfect and better prediction markers are needed for more precise deployment of immunotherapy.

The current practice of PD-L1 expression evaluation has several limitations. Multiple diagnostic anti-PD-L1 antibodies are available. The major clones include 28–8, 22C3, SP142, and SP263. Staining of the same tumor tissue with each of these antibodies may yield different results, which sometimes vary substantially [4]. Another problem is tumor spatial and temporal heterogeneity [5, 6]. PD-L1 expression is not homogenous in lung cancer and the expression level varies with time. Several immunotherapy candidate patients are diagnosed with the disease at advanced stages and the diagnosis is made using needle biopsy specimens obtained from the tumors. It is not surprising that PD-L1 evaluation based on this limited material sometimes fails to reflect the overall PD-L1 expression in the patient’s tumor.

Attempts to overcome heterogeneity in PD-L1 expression include collecting more tissue from the patient, or making more sections for PD-L1 staining from the available tissue. The first approach is not feasible because of the increased invasiveness and potential risk to the patient. The second approach is also ineffective because in this era of precision medicine, there is a need to save the precious tumor specimen for multiple tests to identify the suitable therapy for a particular patient (e.g. epidermal growth factor receptor gene (*EGFR*) mutation analysis for EGFR tyrosine kinase inhibitor therapy). Therefore, any attempt to improve the evaluation of PD-L1 expression in tumor tissue should ideally be no more invasive than the standard biopsy procedure and not consume more patient specimen than a standard immunohistochemitry (IHC) evaluation.

Therefore, our team has developed an innovative solution to this difficult challenge: 3D imaging using a unique, non-destructive tissue-clearing technology, derived from the CLARITY method [7]. Our technology can make 150 μm thick formalin-fixed, paraffin-embedded (FFPE) tissue sections that are transparent to light for a short time. Three-dimensional observation of the tissue and marker quantitation could be achieved by coupling fluorescence-labeled antibody staining with confocal microscopy. This method allows the pathologist to evaluate tumor PD-L1 expression in a much larger population of cells. Moreover, our technology does not destroy the macromolecules in the tissue. Specimens after this imaging process can still be used in other pathological and molecular tests. Thus, this technology can potentially revolutionize tumor PD-L1 expression evaluation.

In this study, we demonstrated the feasibility of three-dimensional PD-L1 quantitation using tissue-clearing technology. We evaluated the variation of PD-L1 expression at different specimen depth levels and correlated the results with clinical response to immunotherapy. We also evaluated the specimen condition after tissue-clearing using various histopathological and molecular tests to demonstrate the preservation of specimen integrity.

## Materials and methods

### Patient population

Patients who were diagnosed with lung adenocarcinoma and underwent surgical resection of primary lung tumor at Taipei Veterans General Hospital from 2012 to 2019 were included in this study. Cases with adenocarcinoma combined with other non-small cell histological components, such as squamous cell carcinoma, were also allowed. Information about their tumor PD-L1 expression level, the diagnostic antibody used in the report, the immunotherapy they received (if any), and the treatment setting (for metastatic disease, as adjuvant or neoadjuvant) were obtained from their medical records. Selected lung adenocarcinoma patients who only received needle biopsy of the primary tumor and who received immunotherapy but showed unexpected clinical response based on their tumor PD-L1 expression were also included in the study. This study was approved by the Institutional Review Board (IRB) of Taipei Veterans General Hospital (No. 2021-01-003C) and was performed in accordance with the Helsinki Declaration. Informed consent has been waived.

### Tissue processing and staining

**Fig. 1:**
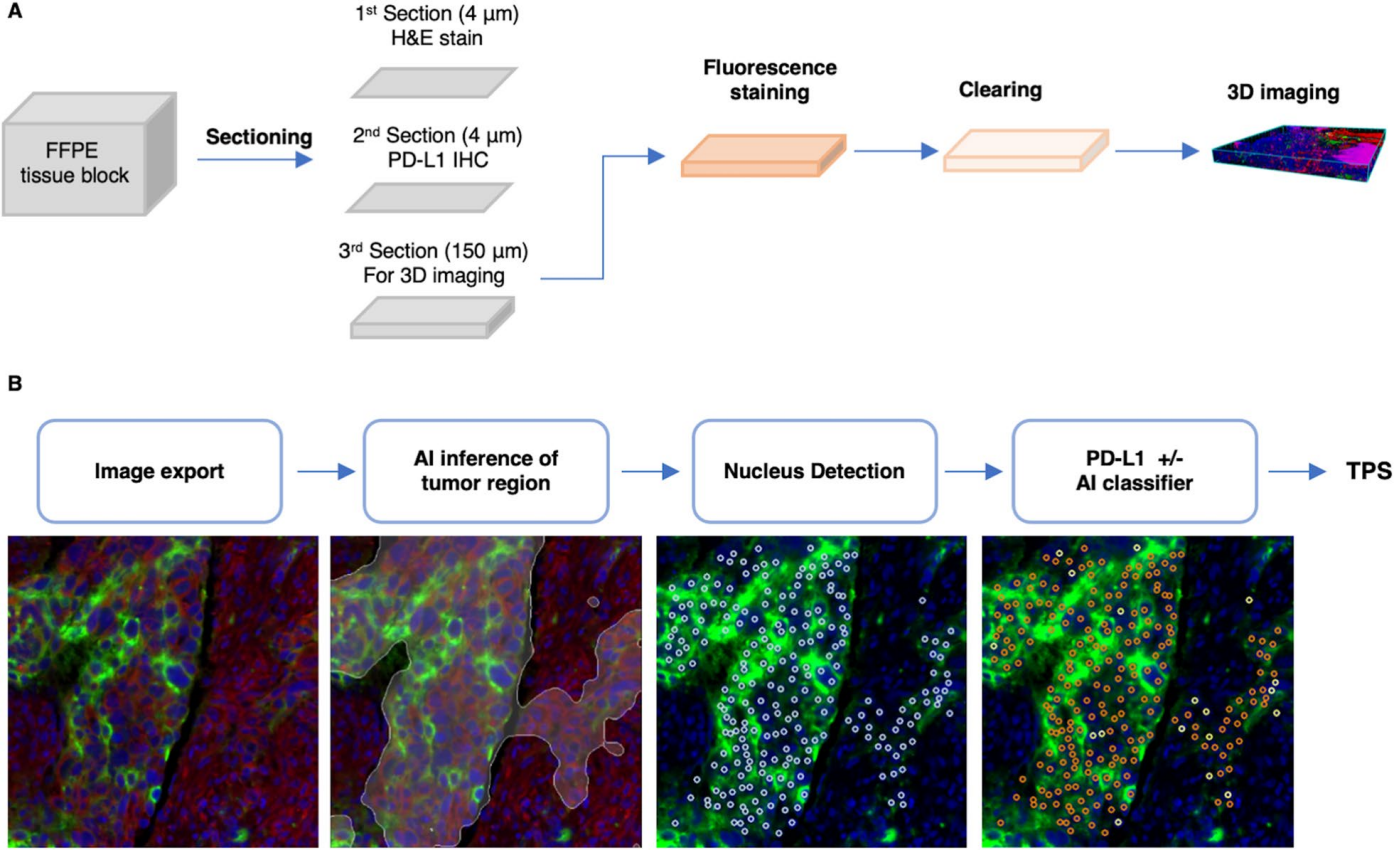
Schematic diagrams of 3D imaging workflow for the assessment of PD-L1 expression of non-small cell lung cancer. **A** Workflow of tissue sectioning, staining, clearing, and imaging for the evaluation of PD-L1 expression in 3D. **B** The structure of artificial intelligence assistance in quantitation of PD-L1 tumor proportion score (TPS). Green: PD-L1; Red: DiD lipid-labeling dye; Blue: SYTO-16 nucleus-labeling dye; White mask: software-inferred tumor region; Orange circle: software-identified PD-L1-positive cell; White circle: software-identified PD-L1-negative cell

The overall workflow is summarized in Fig. 1A. FFPE tissue of the patients’ lung tumors were retrieved from the archives. For surgical resection specimens, one representative tumor block was selected. For needle biopsy specimens, only cases with sufficient material for subsequent thick sectioning were included. Initially, two 4 μm thin sections were made, followed by one 150 μm thick section. The first thin section was subjected to hematoxylin and eosin (H&E) staining. The second thin section was subjected to immunohistochemical staining of PD-L1 using clone SP263 antibody (pre-diluted, Ventana Medical Systems, Oro Valley, AZ, USA). The IHC protocol was modified from manufacturer’s recommendations to match the conditions used in the following immunofluorescence protocol. The sections were de-waxed and subjected to cell conditioning 1 (Tris–EDTA) pretreatment at 100 °C for 1 h. The slides were stained with anti-PD-L1 antibody at 4 °C for 12–16 h. The slides were then treated with the Novolink Min Polymer Detection System (Leica Biosystems, Newcastle, UK). The sections were counterstained with haematoxylin. Human placenta tissue was included as PD-L1 staining control.

Then, the 150 μm thick sections were subjected to immunofluorescence staining and tissue clearing. After dewaxing and rehydration, an 8 mm × 8 mm region of interest was cropped from each section of the surgical resection specimens. This region was selected by a pathologist (YYL) to be a morphological representative. For needle biopsy specimens, all the rehydrated tissue was used for subsequent staining. The specimens were first treated with 2% Triton X-100 (Thermo Fisher Scientific, Waltham, MA, USA) and then incubated with clone SP263 anti-PD-L1 antibody at 4 °C for 12–16 h. The slides were then treated with poly HRP-conjugated goat anti-rabbit Immunoglobulin G (Thermo Fisher Scientific), followed by fluorescence signal amplification with Alexa Fluor 555 Tyramide (Tyramide SuperBoost Kit with Alexa Fluor Tyramines, Thermo Fisher Scientific). The sections were then incubated with lipophilic tracer DiD (20 μg/mL, Thermo Fisher Scientific) and nucleic acid dye SYTO-16 (5 mM, Thermo Fisher Scientific). Finally, the sections were immersed in the clearing reagent (JelloX Biotech Inc., Hsinchu, Taiwan) [8–10] at 20–25 °C overnight. The sections were sealed in the clearing reagent and stored at room temperature before image acquisition.

To use as control, selected specimens after staining, tissue clearing, and image acquisition were dehydrated, embedded in paraffin, and tested for suitability of pathological and molecular examinations, including making 4 μm sections for H&E staining, IHC staining of tissue transcription factor 1 (TTF-1), and DNA extraction for *EGFR* mutation analysis. For TTF-1 IHC on the Leica Bond-Max (Leica Biosystems, Mount Waverley, Australia) automated staining platform, the sections were treated with EDTA buffer (pH 9.0) at 99 °C for 20 min for antigen retrieval and antibody stripping. The sections were stained with anti-TTF-1 antibody (clone 8G7G3/1, 1:300, DAKO, Glostrup, Denmark) at room temperature for 15 min. The slides were then treated with the Bond Polymer Refine Detection Kit (Leica Biosystems, UK) and counterstained with haematoxylin. For *EGFR* mutation analysis, cobas EGFR Mutation Test v2 was performed on a cobas 4800 system (Roche Diagnostics, Basel, Switzerland), according to manufacturer’s protocol. The results were compared to those of the specimens obtained from the same tumor blocks that had not undergone PD-L1 staining or tissue clearing processes.

### Image acquisition and analysis

Whole slide images of the 4 μm thin sections were scanned with a MoticEasyScan scanner (Motic, Hong Kong). The images were viewed by a pathologist (YYL) using Motic Digital Slide Assistant System Lite 1.0 (Motic). The PD-L1 tumor proportion score (TPS) of each case was estimated according to standard clinical practices and expressed as the percentage of tumor cells with any membranous PD-L1 staining among total tumor cells. The score was placed into one of the three categories: < 1%, 1–49%, and ≥ 50%.

For the 150 μm thick sections, 3D fluorescence images were acquired using a spinning disk confocal microscope (Andor Technology, Belfast, UK) with a 20X objective lens. The conditions of laser intensity and exposure time in each channel were fixed. The 488 nm laser intensity was fixed at 30%, and the exposure time was fixed at 15 ms for the SYTO-16 channel. Similarly, the 561 nm laser intensity was fixed at 30%, and the exposure time was fixed at 30 ms for the Alexa Fluor 555 channel. The 637 nm laser intensity was fixed at 15%, and the exposure time was fixed at 15 ms for the DiD channel. Image normalization and export were performed using Imaris 9.7 software (Bitplane, Belfast, UK). The 3D volume visualization was performed using Avizo 9.6 software (Thermo Fisher Scientific). As the fluorescence signal from the staining dye and antibodies is much higher than the tissue autofluorescence in our staining process, the autofluorescence signal did not appear in the images acquired using spinning disk confocal microscope with appropriate exposure settings (Additional file 1: Fig. S1A–J). Compared to the PD-L1 positive case, there is no signal detected in the PD-L1-Alexa-Fluor-555 channel of the PD-L1 negative case (Additional file 1: Fig. S1K–T).

For the surgical resection specimens, although the 3D image of the entire 8 mm × 8 mm × 150 μm processed tissue was obtained, we chose to create a “pseudo-needle biopsy” from this image to simulate application of the technology in needle biopsy specimens. The “pseudo-needle” track was designed to be 8 mm × 1 mm and covering the entire 150 μm in the z-axis. We used the ImageJ software (version 1.53a, National Institute of Health, Bethesda, MD, USA) to determine the center of mass of each 3D image and created the pseudo needle track through this point. This cropped image was then exported using Imaris 9.7 software. For true needle biopsy specimens, entire 3D images were used for subsequent analysis.

The exported images were analyzed using the computer software MetaLite, developed by JelloX Biotech Inc. to determine tumor PD-L1 expression (Fig. 1B and Additional file 1: Fig. S2). The software contains three parts: first, the tumor recognition model generated a tumor area mask to delineate the tumor cells; the software then detected the individual tumor cells based on their nuclear SYTO-16 staining; and finally, the PD-L1 membranous staining AI classifier model determined whether each tumor cell had membranous PD-L1 staining. This allowed calculation of TPS for each image in the X–Y plane.

The tumor segmentation model was developed with a training dataset (30 images) and a testing dataset (10 images) from our cohort. Each two-dimensional (2D) image covering a tissue area of 3 mm × 3 mm in size and containing 5,000–10,000 cells was annotated by trained biologists at JelloX Biotech Inc. and confirmed by a pathologist (YYL) to delineate the tumor area. We designed our model referring to the structure of a benchmarked segmentation model High-Resolution Network version 2 (HRNetV2) [11]. The network model was implemented on the NVIDIA GTX2080 ti GPU using TensorFlow architecture. We adopted cell instance accuracy to evaluate the performance of our model. By applying the following nuclear segmentation algorithm to the testing set, we determined how many software-predicted tumor cells were also the pathologist-confirmed tumor cells.

The nuclear segmentation algorithm was built based on previous methods [12, 13, 14]. Using the SYTO-16 channel, we applied the Li-threshold algorithm to remove background, fill foreground holes, and find connected components, obtaining rough nuclei labels. Subsequently, using the watershed algorithm on the local maxima image, we separated neighboring nuclei, obtaining the position of each nucleus and the total number of nuclei. The performance of this algorithm was evaluated using the BSST265 dataset [15]. This dataset consists of 79 fluorescence images containing 7813 nuclei.

To determine whether each tumor cell recognized by the above models had membranous PD-L1 staining, we built the PD-L1 membranous staining AI classifier model. A total of 85 fluorescence images (containing 9822 cells) from our patient cohort were collected for training. Another 10 images from the cohort containing 2,544 cells were prepared for testing. Both image sets were reviewed by two pathologists (YYL and LCW) using an auxiliary software (Additional file 1: Fig. S2B). Each cell in the images was given a positive/negative label. Each pathologist annotated the image sets twice, with a washout period of 2 weeks. A cell would be classified as PD-L1-positive only if it was annotated positive four times. We used a CNN-based classification model to process the training data (Additional file 1: Fig. S2C, D) using the same hardware and architecture as the tumor segmentation model [16]. The ability of the model to correctly identify cells with PD-L1 membranous staining was evaluated using the testing set.

**Fig. 2:**
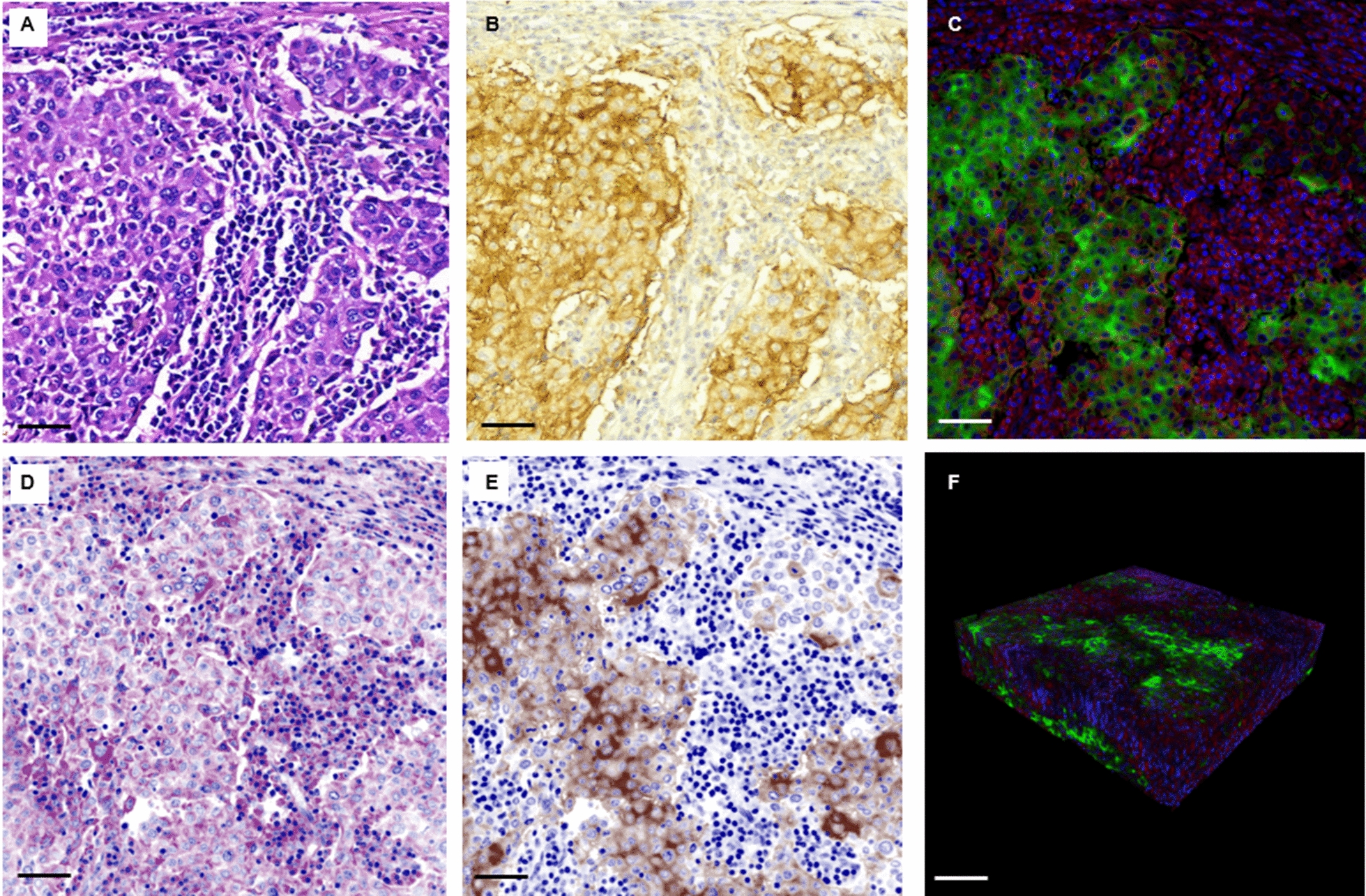
High resolution image of non-small cell lung cancer PD-L1 expression from formalin-fixed, paraffin-embedded specimens generated using immunofluorescence staining and tissue clearing. **A**, **B** The standard H&E staining and PD-L1 immunohistochemistry (IHC) performed on 4 μm sections of one representative case, respectively. The PD-L1 immunofluorescence and tissue clearing protocols produced image (**C**) was comparable to the traditional IHC, including the membranous staining pattern of PD-L1. The fluorescence image can be converted to pseudo-H&E (**D**) and pseudo-IHC (**E**) formats for assessment by pathologists. The three-dimensional projection of the immunofluorescence image (**F**) shows good tissue details of the entire specimen (Green: PD-L1, red: DiD, and blue: SYTO-16. Scale bar: A–E: 50 μm, F: 150 μm)

We then used the complete model to calculate TPS of all cases in our cohort. The TPS of each X–Y plane was plotted against the tissue depth (z-axis) at 5 μm interval, and the average TPS of the entire tissue in 3D was calculated. The final TPS score was also placed into one of the three categories: < 1%, 1–49%, and ≥ 50%. As a final validation of our algorithm, we selected one 2D fluorescence image from each case and compared the TPS generated by the algorithm with the TPS visually estimated by two pathologists (YYL and LCW).

### Statistical analysis

We used weighted kappa to determine the concordance of TPS categorization between the original pathology report and the TPS generated by viewing IHC staining of 4 μm thin sections based on our modified protocol. We used the same method to determine the concordance between TPS derived from IHC and TPS derived from viewing the superficial layer of our 3D immunofluorescence images. We also used this method to compare the concordance between artificial intelligence-determined TPS and pathologist’s TPS estimation based on immunofluorescence images. A kappa value greater than 0.6 was considered good and a value greater than 0.8 was considered excellent.

## Results

### Patient characteristics

During the study period, 33 patients with surgical resection specimens fulfilling the inclusion criteria were identified. The basic clinical and pathological information of the selected patients is summarized in Table 1. Among the 33 cases, 30 cases were previously tested using the 22C3 clone of anti-PD-L1 antibody, one using clone SP142, and two using clone SP263. Twenty-two patients were subsequently subjected to immunotherapy. Since the patients were treated with immunotherapy under various clinical scenarios, with no limitation of concurrent treatment modalities, the correlation between pathological PD-L1 scoring and treatment response could not be easily evaluated. Nineteen patients were treated for stage IV metastatic disease. Two were treated in an adjuvant setting, while one was treated in a neoadjuvant setting. The clinical features of the two studied patients from whom needle biopsy specimens were collected are described individually later in the article.

**Table 1:** Characteristics of patients in this study

Total number of patients	33
Mean age (Range)	63.7 (39–83)
Sex (Male:Female)	18:15
Clinically reported PD-L1 score	
Based on clone 22C3	
TPS < 1%	10
TPS 1–49%	14
TPS ≥ 50%	6
Based on clone SP142	1 (TC 0%, IC 15%)
Based on clone SP263	
TPS < 1%	1
TPS 1–49%	0
TPS ≥ 50%	1
Number of patients who Received Immunotherapy	22
Pembrolizumab	9
Nivolumab	6
Atezolizumab	7
Treatment Scenario	
Stage IV Disease	19
Adjuvant	2
Neoadjuvant	1

*PD-L1* programmed death-ligand 1, *TPS* tumor proportion score, *TC* tumor cell, *IC* immune cell

### PD-L1 expression can be demonstrated in 3D after immunofluorescence staining of FFPE tissue and optical-clearing

As shown in Fig. 2A–C, high resolution image could be generated from the 150 μm thick section of lung cancer tissue. The quality of the image was comparable to that of the traditional H&E staining and PD-L1 immunohistochemistry performed on thin sections. The membranous staining pattern of PD-L1 was observed. The fluorescence image could also be converted to pseudo-H&E and IHC color for pathologists to evaluate in a familiar format (Fig. 2D, E). In the three-dimensional view (Fig. 2F), the image quality was homogenous in the entire tissue. Comparison between the top, middle, and bottom levels of the processed tissue (Additional file 1: Fig. S3A-C) showed equal clarity in cellular details.

As we modified the anti-PD-L1 antibody staining protocol to allow the staining of the thicker sections, we verified our protocol by staining 4 μm thin sections of human placenta tissue followed by 3,3’-Diaminobenzidine histochemistry. The result was the expected standard pattern (Additional file 1: Fig. S3D). The trophoblasts were stained positive, whereas the villous stromal cells and blood vessels were stained negative. A comparison was made between the PD-L1 TPS derived from the 4 μm thin sections stained with modified IHC protocol and the TPS originally reported clinically using standard protocol (excluding one case stained with clone SP142 in the original report, which is known to behave differently from other anti-PD-L1 antibodies [4]). Twenty-four of the 32 cases (75%) had a TPS in the same category (Additional file 1: Fig. S3E). The weighted kappa value was 0.7718. These results indicate that our modified anti-PD-L1 staining protocol did not introduce bias in PD-L1 staining.

Similarly, a comparison between the PD-L1 TPS derived from the 4 μm thin sections stained with the modified IHC protocol and the TPS derived from viewing the top layer of the 150 μm thick sections stained with the immunofluorescence protocol was made and 29 of the 33 cases (87.9%) had a TPS in the same category (Additional file 1: Fig. S3F). The weighted kappa value was 0.7022. This level of agreement was considered good; in the majority of cases, switching to a fluorescence staining/imaging format did not cause a bias in PD-L1 scoring.

### Computer-assisted quantitation of PD-L1 expression in three-dimensional space is accurate and shows significant variation across tissue depth levels

We developed a computer software to analyze the stacks of 2D images contained in each 3D image file. Each layer of the PD-L1 staining fluorescence image was processed by our tumor recognition AI model to generate the tumor mask (Additional file 1: Fig. S4A–C). Individual tumor cells were recognized, then our PD-L1 membranous staining AI classifier model determined their PD-L1 positive/negative status and calculated the PD-L1 TPS (Additional file 1: Fig. S4D–F). Our validation process showed that both the tumor recognition AI model and the PD-L1 membranous staining AI classifier model showed an accuracy greater than 90% (Additional file 1: Tables S1, S2). Our nuclei segmentation algorithm generated an average error of 8% and a standard deviation of 4.9% in 79 fluorescence images of a reference dataset [15]. We further compared the AI-determined TPS with the estimations of pathologists using one representative 2D PD-L1 immunofluorescence image from each of the 33 cases studied. The AI-calculated TPS was concordant with pathologist-assessed TPS in 87.9% (YYL) and 69.7% (LCW) of cases, with weighted kappa value of 0.8299 and 0.4795, respectively, which is a remarkable performance based on this small set of training material (Additional file 1: Table S3).

**Fig. 3:**
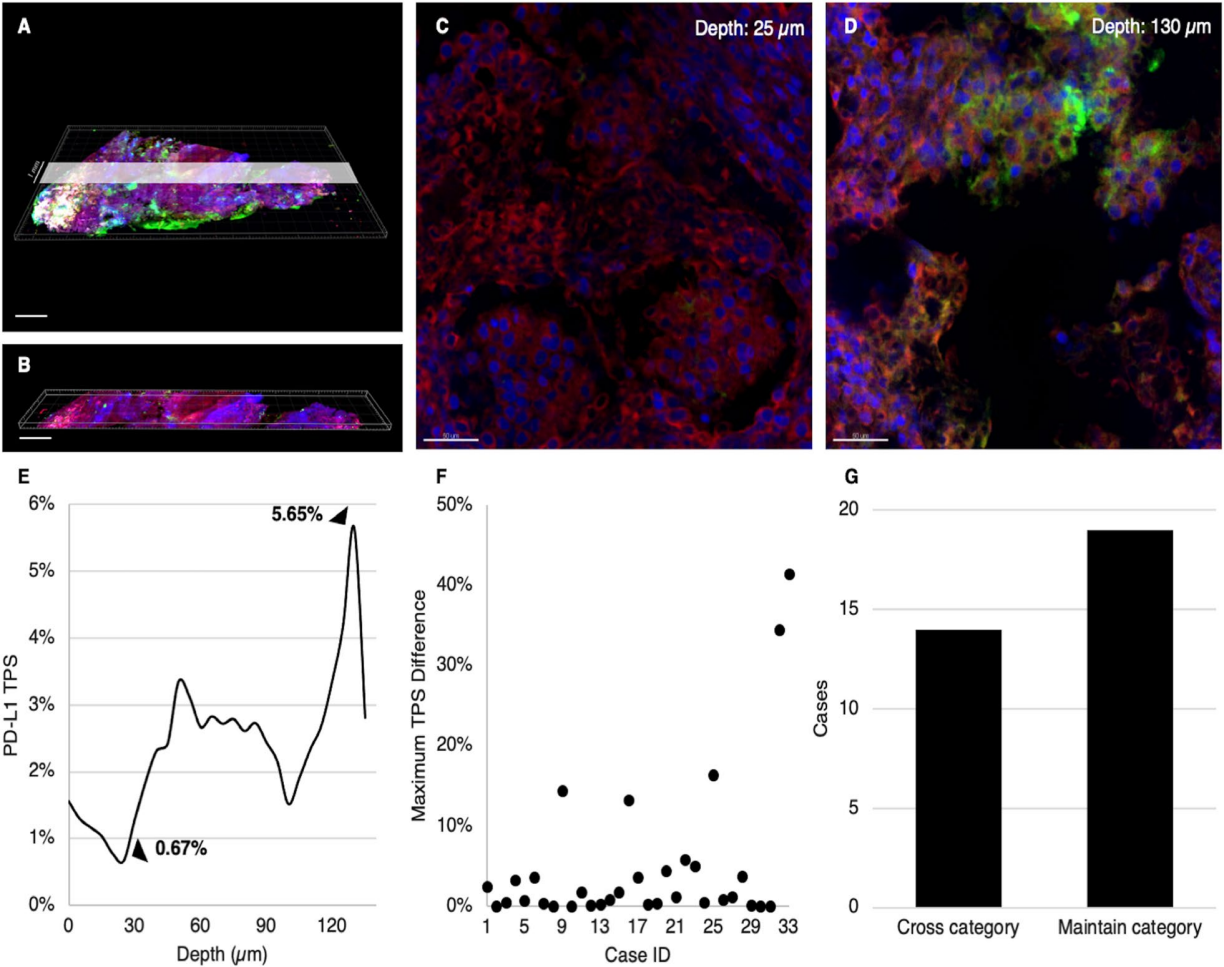
Computer-assisted quantitation of PD-L1 expression in 3D space shows clinically significant variation at different tissue depth levels. **A**, **B** To simulate the main future application of this technology in needle biopsy specimens, we generated pseudo-needle biopsy images that are 8 mm in length, 1 mm in width, and 150 μm in depth from the 3D images obtained from surgical resection specimens. **C**–**E** In this limited volume of specimen, the PD-L1 TPS varied significantly at different tissue depth levels, as shown in one representative case. **F** The average difference between the highest and lowest TPS observed in the pseudo-needle biopsy 3D images was 4.9%, with 5 out of 33 cases (15.2%) showing a TPS difference greater than 10%. **G** In 14 of 33 cases (42.4%), the TPS fell into different categories at different tissue depth levels, while in 19 cases (57.6%) the TPS category was constant throughout the specimen (Scale bar: A–B: 1000 μm, C–D: 50 μm)

We then applied the computer algorithm to the “pseudo-needle biopsy” images of our 33 cases to calculate 3D PD-L1 TPS (Fig. 3A, B). In this limited volume of specimen simulating real needle biopsies, we observed that the PD-L1 TPS varied significantly at different tissue depth levels, as shown in one representative case (Fig. 3C–E). The average difference between the highest and lowest TPS observed in each pseudo-needle biopsy 3D image was 4.9%, with 5 of 33 cases (15.2%) showing a TPS difference greater than 10% (Fig. 3F). Fourteen of 33 cases (42.4%) showed TPS values at different depth levels that belonged to different TPS categories (Fig. 3G). Twelve cases showed a TPS below 1% at certain levels but above 1% at other levels, whereas two case showed a TPS below 50% at certain levels but above 50% at other levels (the TPS-depth relationship of each case is demonstrated in Additional file 1: Fig. S5, and the corresponding detailed clinical and pathological information for each individual case is listed in Additional file 1: Table S4). These results highlight the significance of evaluating PD-L1 expression at different tissue depth levels.

### Difference between 3D PD-L1 score and single thin section PD-L1 score may explain unexpected immunotherapy outcome in selected cases

**Fig. 4:**
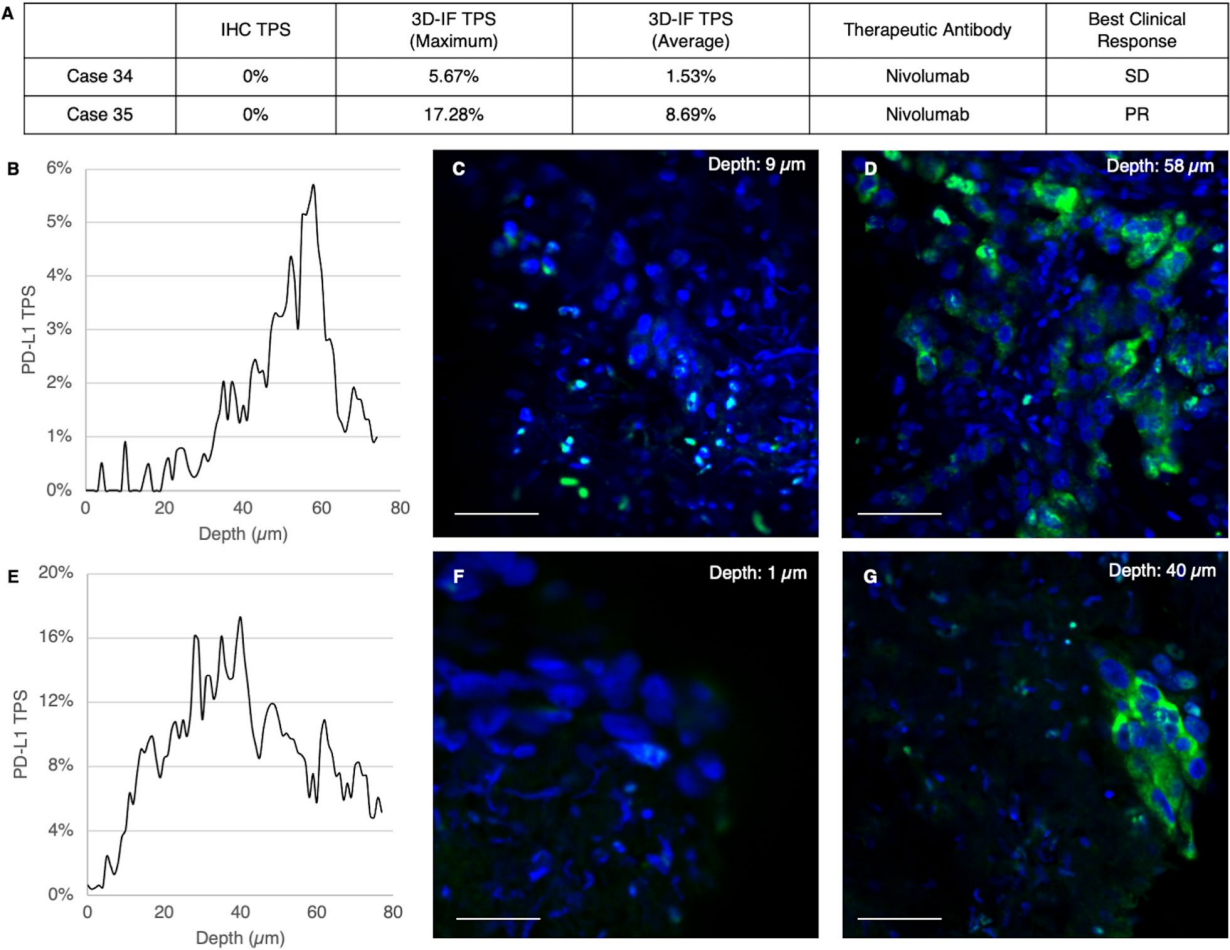
Application of 3D PD-L1 scoring method to actual non-small cell lung cancer needle biopsy specimen shows difference compared to originally reported TPS and may explain unexpected immunotherapy treatment responses. **A** Two lung cancer patients with 0% tumor PD-L1 expression as evaluated by the standard immunohistochemistry method achieved stable disease or partial response under nivolumab treatment. **B**–**D** 3D PD-L1 expression analysis of case 34 showed higher tumor cell PD-L1 expression at deeper levels, reaching 5.67%, above the 1% cut-off value. **E**–**G** Analysis of case 35 also showed higher tumor cell PD-L1 expression at deeper levels, up to 17.28% (SD: stable disease; PR: partial response; IF: immunofluorescence. Green: PD-L1 and blue: SYTO-16. Scale bar: 50 μm)

We next sought to apply our technology to actual needle biopsy specimens. We chose two cases with apparent discrepancy between tumor tissue PD-L1 expression and clinical response to immunotherapies (Fig. 4A). Patient number 34 was a 69-year-old woman who had lung adenocarcinoma with brain metastasis. She received nivolumab treatment and her lung tumor size remained stable for 5 months. The PD-L1 TPS of her lung cancer biopsy specimen was 0%. Patient number 35 was a 65-year-old man with stage IIIA lung adenocarcinoma who received definitive chemoradiation therapy. He suffered from tumor recurrence in neck lymph nodes and received treatment with nivolumab and partial response of the tumor was achieved, lasting for six months. The PD-L1 TPS of his lung cancer biopsy specimen was 0%. It is known that treatment response to nivolumab, a therapeutic anti-PD-1 antibody, does not depend on tumor expression of PD-L1 [17]. However, we suspected that 3D imaging of these two patients’ specimens may reveal PD-L1 expression at deeper depth levels and this may contribute to their disease stabilization or response to immunotherapy.

Indeed, by analyzing 150 μm thick sections, we discovered that both their PD-L1 TPS were not zero: patient number 34 showed an average TPS of 1.53%, with a maximum of 5.67%. Patient number 35 showed an average TPS of 8.69%, with a maximum of 17.28%. The TPS-depth curve and representative images (Fig. 4B–G) suggested that although the superficial layers of the biopsy tissue showed very low PD-L1 expression, substantially higher expression was observed at deeper levels. These two cases demonstrated the potential value of 3D PD-L1 expression evaluation in the guidance of cancer immunotherapy.

### Tissue after processing for three-dimensional imaging is still suitable for histopathological and molecular examinations

**Fig. 5:**
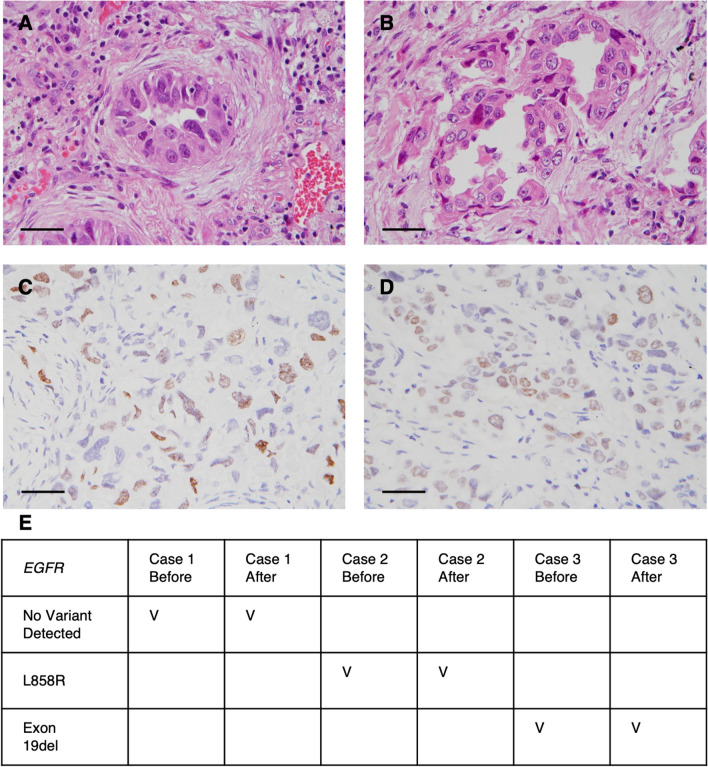
Specimen after 3D imaging could be recovered for standard pathology examinations for non-small cell lung cancer. Comparing the pre-processing (**A**) and post-processing (**B**) tissue, the standard H&E staining performed on 4 μm sections showed similar morphological details. The pre-processing (**C**) and post-processing (**D**) tissue also showed comparable TTF-1 staining by immunohistochemistry. The *EGFR* mutation analysis performed using pre- and post-processing tissue yielded identical results in all the tested cases (n = 3) (**E**) (Scale bar: 40 μm)

After 3D imaging, we re-embedded the 150 μm thick tissue sections into paraffin blocks and high-quality H&E-stained tissue sections could be made from the re-embedded tissue (Fig. 5A, B). The immunohistochemical staining of TTF-1 was also comparable to that of the routine sections (Fig. 5C, D). *EGFR* mutation tests performed on the DNA extracted from the re-embedded tissue showed 100% concordance with the tests performed on original FFPE tissue (Fig. 5F). These results indicate that the tissue clearing and three-dimensional imaging processes preserve important tissue morphological details and molecular compositions.

## Discussion

In this study, we demonstrated that evaluation of non-small cell lung cancer PD-L1 expression in 3D was applicable to FFPE tissue. We showed significant variation in PD-L1 expression at different tissue depth levels, and the PD-L1 expression in 3D may correlate with clinical response to immune checkpoint blockade therapy in selected cases. Moreover, our technology preserved specimen integrity, allowing the processed tissue to be used for other important histopathological and molecular tests. We believe this method has the potential to replace traditional 2D PD-L1 expression evaluation in the future.

Our unique tissue-clearing technology is the foundation of this approach. The tissue is made optically transparent by immersion inthe solution with same refractive index. Our technology does not require the use of detergents such as sodium dodecyl sulfate, digestive enzymes, or structure-supporting polymers, preserving the native macromolecules in the specimen. These advantages are crucial in the evaluation of PD-L1 expression. Compared to the 3D PD-L1 evaluation method developed by Korehisa et al. [18], which was based on computer reconstruction of PD-L1 IHC image from serial thin sections, our method has the advantages of “true” 3D imaging and preserved tissue integrity after imaging for further pathological examinations. Compared to the method developed by Lee et al*.* [19], which required embedding lightly-fixed tumor tissue in agarose gel for subsequent processing, our method can be used on FFPE tissue. Therefore, our method can be more easily incorporated into routine pathology laboratory practices.

Due to the limited sample size of this study, we could not determine whether 3D PD-L1 expression scores correlate better with immune checkpoint blockade therapy outcome than 2D scores. Prospective studies with proper control of the patient condition, type of immunotherapy used, type of concurrent treatment modalities, and adequate follow-up period will be required to answer this question. How 3D PD-L1 score correlates with other predictive biomarkers for immunotherapy response, such as tumor mutation burden or microsatellite instability [20] is another crucial question. Importantly, all 33 surgical resection specimens we studied consisted mainly of adenocarcinoma. One case also contained a squamous cell carcinoma component, one contained a large cell neuroendocrine component, and two cases fulfilled the criteria for pleomorphic carcinoma. Therefore, our PD-L1 quantitation algorithm has limited challenge from non-adenocarcinoma histology types. In addition, the concordance of fluorescence image PD-L1 TPS between computer calculation and pathologist estimation could still be improved. More cases and more pathologists need to be involved to improve the algorithm. Our proof-of-concept experiments showed the post-tissue clearing specimen was still suitable for a variety of pathological assessments such as H&E staining, immunohistochemistry staining, and *EGFR* genotyping. The exact range of applications that can be accommodated, as well as the pre-/post-clearing result concordance rate in a larger number of cases, will need to be determined before the technology can be widely accepted to be not interfering normal pathology specimen stewardship.

The 3D image of tumor tissue can provide us with more information than just tumor PD-L1 expression. For example, in lung adenocarcinoma, a lepidic growth pattern with thick septum may appear very similar to acinar pattern in thin sections. Three-dimensional image analysis may help define these growth patterns more clearly. The current study focused on the tumor cell expression of PD-L1. However, PD-L1 expression by immune cells is also important in a patient’s outcome and immune cell expression of PD-L1 is already included in the assessment of clone SP142 anti-PD-L1 diagnostic antibody staining [21]. Quantitating PD-L1 expression in three-dimensional space, with further differentiation between tumor cell and immune cell, is an important next step. Moreover, understanding the spatial distribution of the immune cells in 3D may also provide us with new insights into tumor immune microenvironment. In addition to application in lung cancer diagnosis, our technology can also potentially be applied to the evaluation of markers in other diseases. For example, the evaluation of HER2 expression in breast cancer cells also specifically requires the recognition of membranous staining. Three-dimensional imaging of breast cancer specimens may also address tumor heterogeneity in HER2 expression and contribute to accurate pathological assessment of the specimen and precise deployment of anti-HER2 therapies. In patients without the availability of large resection specimens, computer-assisted, three-dimensional biomarker scoring would be an alternative option to evaluate the limited biopsy specimens in a more accurate way in the era of precision and personalized medicine. With the advent of mature tissue-clearing technology and imaging methods, the impact of 3D pathology is just beginning to unfold and ever-broader application of the technology will be the foundation of new discoveries.

## Supplementary Information

**Additional file 1.** **Fig. S1.** The comparison of autofluorescence signal and immunofluorescence staining signal in formalin-fixed, paraffin-embedded tissue of non-small cell lung cancer. (**A**–**J**) In the images captured from two control samples without immunofluorescence staining, all of the channels revealed low signal. As the fluorescence signal from staining dye and antibodies is much higher than the tissue autofluorescence, the autofluorescence signal did not appear in the images acquired using spinning disk confocal microscope with appropriate exposure settings. (**K**–**T**) Representative fluorescence images of one PD-L1-positive case and one PD-L1- negative case were shown here. Compared to the PD-L1 positive case, there is no signal detected in the PD-L1- Alexa-Fluor-555 channel of the PD-L1 negative case. **Fig. S2.** Schematic diagrams of the development of artificial intelligence (AI)-assisted PD-L1 expression quantitation. The AI model consists of three parts: a lung tumor segmentation model, a nucleus segmentation model, and a PD-L1 membranous staining classification model. The architecture of the lung tumor segmentation model is outlined in (**A**). The network contains three parts; the details of each part are shown in (**D**), including: part A, Bottleneck block, which is stronger for maintaining the spatial resolution and enlarging the receptive field; part B: Basic block, which is better in maintaining low-level features; part C: Conv block, which is the basic unit of the network composed of a convolutional layer, batch normalization layer, and ReLU (Rectified Linear Units) activation function. In the development of the PD-L1 membranous staining classification model, we used an auxiliary software shown in (**B**) to allow pathologists to annotate the PD-L1 membranous staining status of cells in the training material. This information was used to train the PD-L1 membranous staining classification model, the architecture of which is shown in (**C**), which also shares the structure details of the AI models shown in (**D**). **Fig. S3.** PD-L1 immunofluorescence staining and tissue clearing produce images of stable quality at different depth levels of the specimen and perform comparably to the standard PD-L1 immunohistochemistry. Representative 2D images from the superficial (**A**), middle (**B**) and deep (**C**) layers of the 3D fluorescence image of one specimen show equally good quality in tissue details. (**D**) Applying the same anti-PD-L1 antibody staining condition to 4 μm sections of human placenta tissue followed by histochemistry resulted in the expected staining pattern. (**E**) Applying the same condition to 4 μm sections of 32 lung cancer specimens followed by histochemistry produced PD-L1 TPS comparable to the original TPS reported clinically. One case was excluded from the analysis because the original report was based on clone SP142 anti- PD-L1 antibody, and it is known to behave differently from clone SP263 used in this study. (**F**) The TPS obtained from examination of the superficial layer of the immunofluorescence (IF) image of each specimen is mostly concordant with its TPS obtained from the immunohistochemistry of adjacent 4 μm sections. (Scale bar: 100 μm) **Fig. S4.** The performance of the tumor recognition AI model and the PD-L1 membranous staining classification model. (**A**) Representative fluorescence image of a tumor specimen. (**B**) The AI-recognized tumor area is shown in the white mask. (**C**) Compared to pathologist’s annotation, the AI recognition is mostly correct. Green: true positive. Blue: true negative. Yellow: false positive (not tumor but recognized as tumor by AI). Red: false negative (tumor but recognized as not tumor by AI). (**D**) Representative fluorescence image with PD-L1 staining in green. (**E**) Nuclei detection by nuclei segmentation. In this image, it is not limited to the tumor area. (**F**) Result of PD-L1 membranous staining classification. Orange circle: PD-L1 positive. White circle: PD-L1 negative. (Scale bar: **A**–**C**: 500 μm, **D**–**F**: 100 μm) **Fig. S5.** Relationship between PD-L1 TPS and tissue depth in all 33 pseudo-needle biopsy lung cancer specimens. (**A**) The PD-L1 TPS of each case across different tissue depth levels at 5 μm interval. Due to rehydrated section thickness variability, not every case has the same total depth analyzed. (**B**) The same data as shown in (**A**), excluding the five cases with TPS higher than 10%, highlighting the TPS variation in the lowexpression cases. (**C**) Box plot of TPS of each case across the analyzed tissue depth, showing the median (-), average (x), interquartile range, maximal and minimal value. (**D**) The same data as shown in (**C**), excluding the five cases with TPS higher than 10%, highlighting the TPS variation in the low-expression cases.

## Data Availability

The datasets used and analyzed during the current study are available from the corresponding author upon reasonable request.

## Abbreviations

2D: Two-dimensional
3D imaging: Three-dimensional imaging
EGFR: Epidermal growth factor receptor gene
FFPE tissue: Formalin-fixed, paraffin-embedded
H&E: Hematoxylin and eosin
IHC: Immunohistochemistry
PD-L1: Programmed death-ligand 1
TPS: Tumor proportion score
TTF1: Tissue transcription factor 1

## Code availability

The code used during the current study is available from the corresponding author upon reasonable request.
