# Supplementary material for: Computer-assisted three-dimensional quantitation of programmed death-ligand 1 in non-small cell lung cancer using tissue clearing technology
Source: J Transl Med. 2022 Mar 16;20:131. doi: 10.1186/s12967-022-03335-5 (PMC8925228; doi:10.1186/s12967-022-03335-5)
Supplement: Supplementary file 1 — Additional file 1. Fig. S1. The comparison of autofluorescence signal and immunofluorescence staining signal in formalin-fixed, paraffin-embedded tissue of non-small cell lung cancer. (A–J) In the images captured from two control samples without immunofluorescence staining, all of the channels revealed low signal. As the fluorescence signal from staining dye and antibodies is much higher than the tissue autofluorescence, the autofluorescence signal did not appear in the images acquired using spinning disk confocal microscope with appropriate exposure settings. (K–T) Representative fluorescence images of one PD-L1-positive case and one PD-L1- negative case were shown here. Compared to the PD-L1 positive case, there is no signal detected in the PD-L1- Alexa-Fluor-555 channel of the PD-L1 negative case. Fig. S2. Schematic diagrams of the development of artificial intelligence (AI)-assisted PD-L1 expression quantitation. The AI model consists of three parts: a lung tumor segmentation model, a nucleus segmentation model, and a PD-L1 membranous staining classification model. The architecture of the lung tumor segmentation model is outlined in (A). The network contains three parts; the details of each part are shown in (D), including: part A, Bottleneck block, which is stronger for maintaining the spatial resolution and enlarging the receptive field; part B: Basic block, which is better in maintaining low-level features; part C: Conv block, which is the basic unit of the network composed of a convolutional layer, batch normalization layer, and ReLU (Rectified Linear Units) activation function. In the development of the PD-L1 membranous staining classification model, we used an auxiliary software shown in (B) to allow pathologists to annotate the PD-L1 membranous staining status of cells in the training material. This information was used to train the PD-L1 membranous staining classification model, the architecture of which is shown in (C), which also shares the structure d [file 12967_2022_3335_MOESM1_ESM.docx]

**
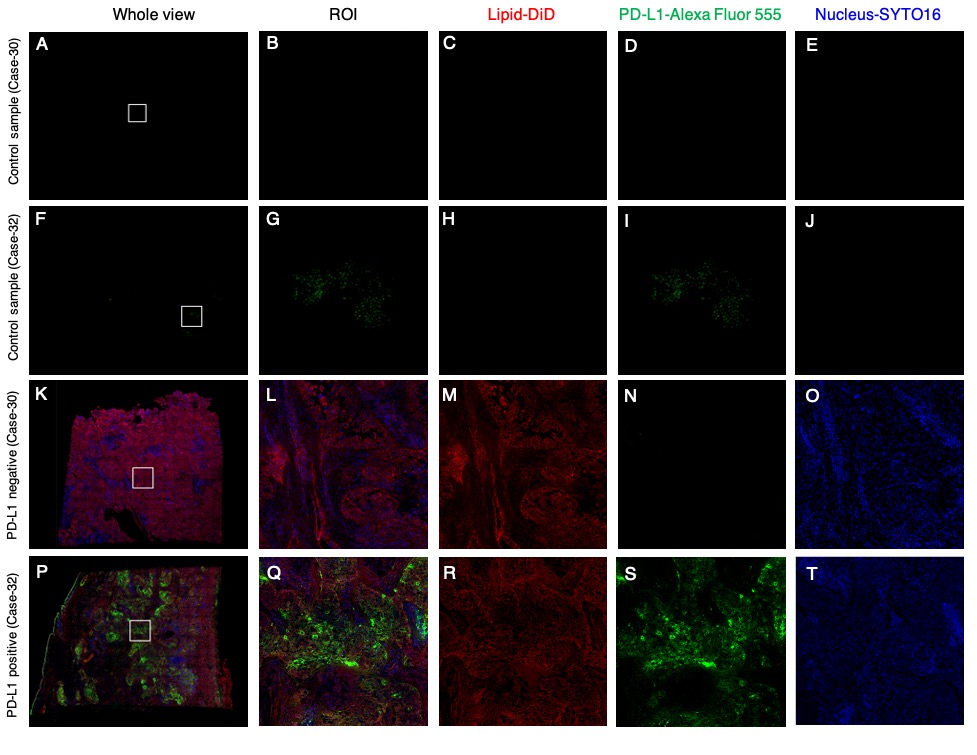
**

**Fig. S1. The comparison of autofluorescence signal and immunofluorescence staining signal in formalin-fixed, paraffin-embedded tissue of non-small cell lung cancer.** (A-J) In the images captured from two control samples without immunofluorescence staining, all of the channels revealed low signal. As the fluorescence signal from staining dye and antibodies is much higher than the tissue autofluorescence, the autofluorescence signal did not appear in the images acquired using spinning disk confocal microscope with appropriate exposure settings. (K-T) Representative fluorescence images of one PD-L1-positive case and one PD-L1-negative case were shown here. Compared to the PD-L1 positive case, there is no signal detected in the PD-L1-Alexa-Fluor-555 channel of the PD-L1 negative case.


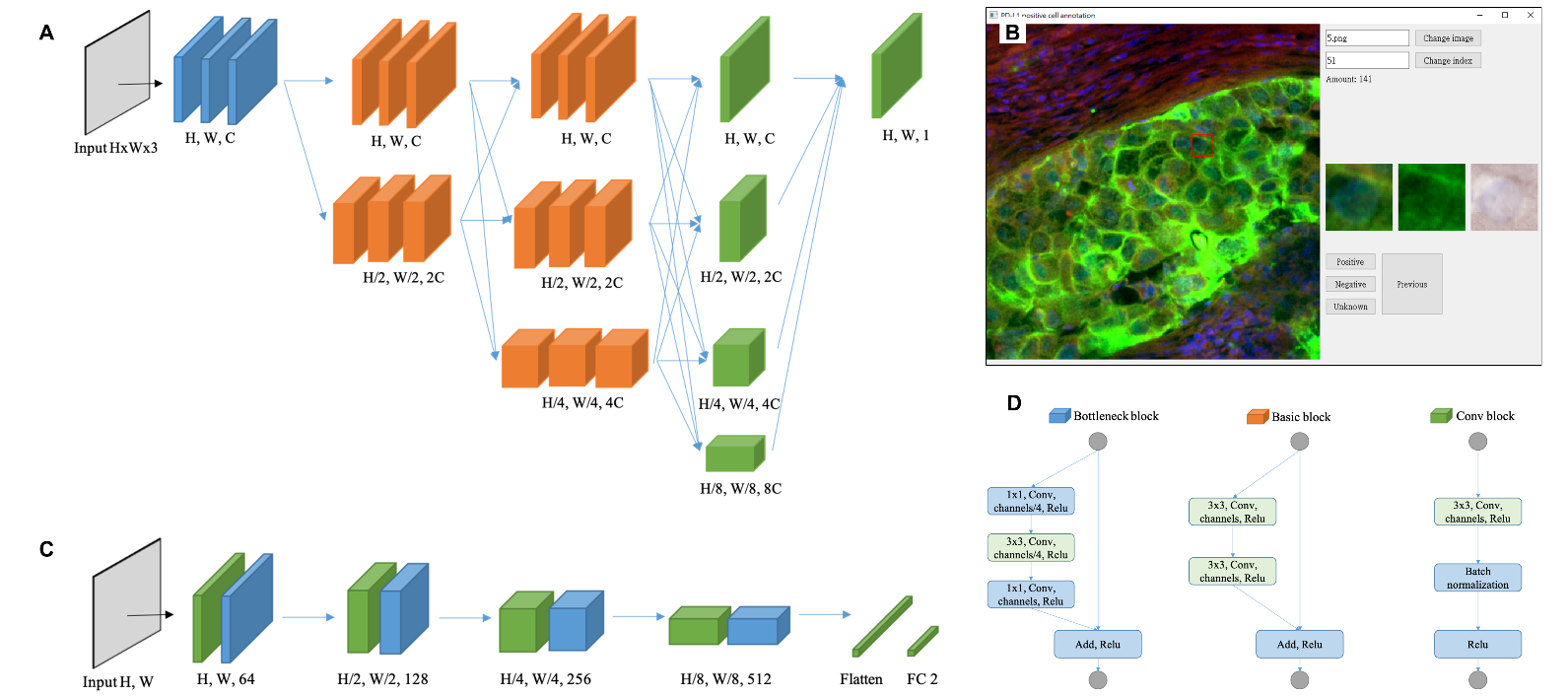


**Fig. S2. Schematic diagrams of the development of artificial intelligence (AI)-assisted PD-L1 expression quantitation.**

The AI model consists of three parts: a lung tumor segmentation model, a nucleus segmentation model, and a PD-L1 membranous staining classification model. The architecture of the lung tumor segmentation model is outlined in **(A)**. The network contains three parts; the details of each part are shown in **(D)**, including: part A, Bottleneck block, which is stronger for maintaining the spatial resolution and enlarging the receptive field; part B: Basic block, which is better in maintaining low-level features; part C: Conv block, which is the basic unit of the network composed of a convolutional layer, batch normalization layer, and ReLU (Rectified Linear Units) activation function. In the development of the PD-L1 membranous staining classification model, we used an auxiliary software shown in **(B)** to allow pathologists to annotate the PD-L1 membranous staining status of cells in the training material. This information was used to train the PD-L1 membranous staining classification model, the architecture of which is shown in **(C)**, which also shares the structure details of the AI models shown in **(D)**.


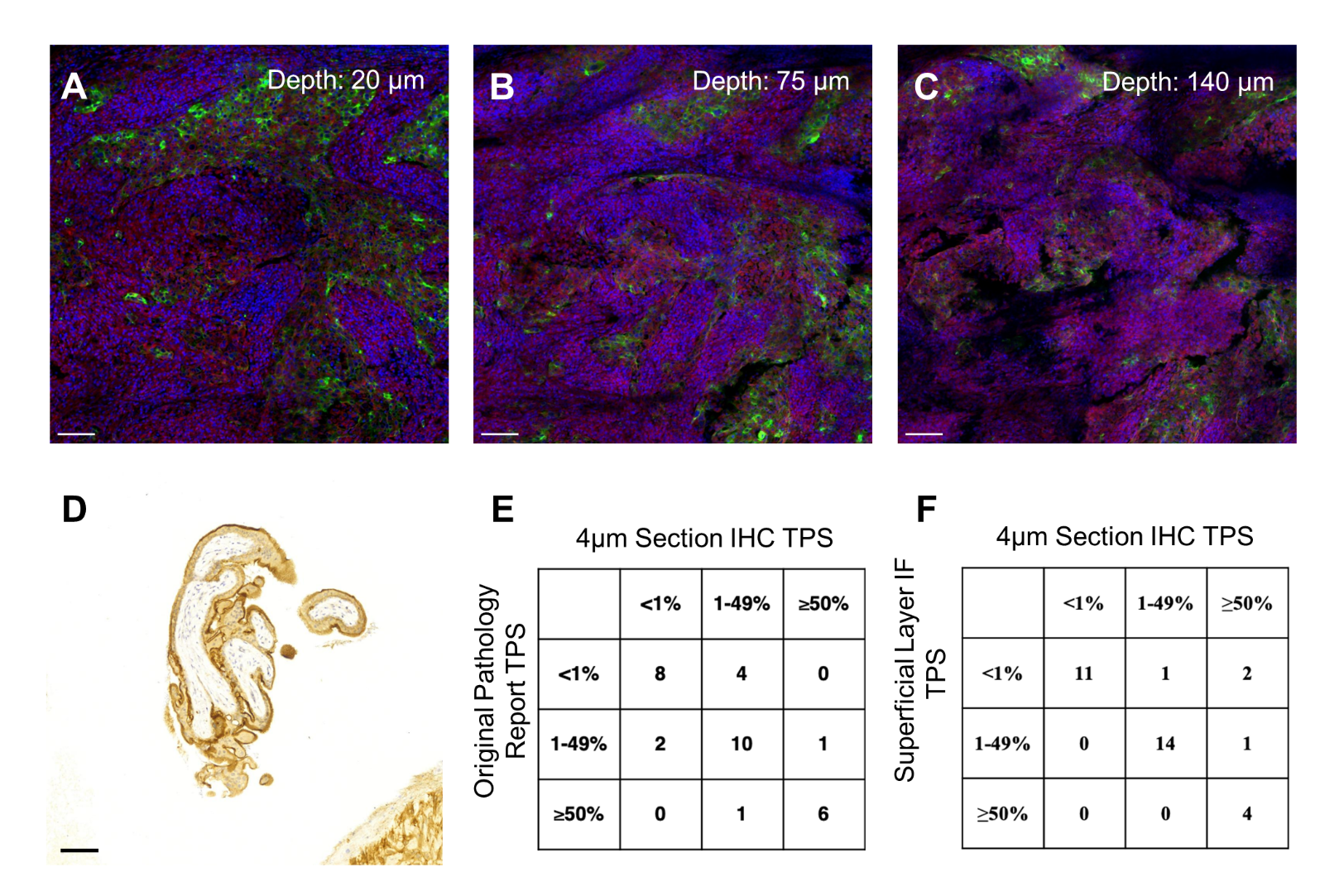


**Fig. S3. PD-L1 immunofluorescence staining and tissue clearing produce images of stable quality at different depth levels of the specimen and perform comparably to the standard PD-L1 immunohistochemistry.**

Representative 2D images from the superficial **(A)**, middle **(B)** and deep **(C)** layers of the 3D fluorescence image of one specimen show equally good quality in tissue details. **(D)** Applying the same anti-PD-L1 antibody staining condition to 4 μm sections of human placenta tissue followed by histochemistry resulted in the expected staining pattern. **(E)** Applying the same condition to 4 μm sections of 32 lung cancer specimens followed by histochemistry produced PD-L1 TPS comparable to the original TPS reported clinically. One case was excluded from the analysis because the original report was based on clone SP142 anti-PD-L1 antibody, and it is known to behave differently from clone SP263 used in this study. **(F)** The TPS obtained from examination of the superficial layer of the immunofluorescence (IF) image of each specimen is mostly concordant with its TPS obtained from the immunohistochemistry of adjacent 4 μm sections. (Scale bar: 100 μm)


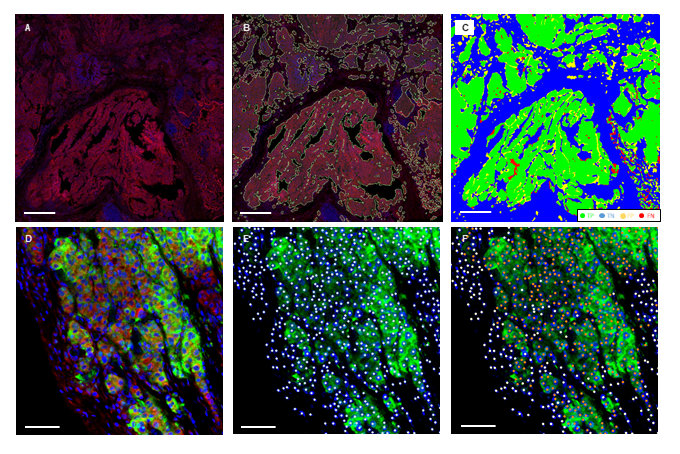


**Fig. S4. The performance of the tumor recognition AI model and the PD-L1 membranous staining classification model.**

**(A)** Representative fluorescence image of a tumor specimen. **(B)** The AI-recognized tumor area is shown in the white mask. **(C)** Compared to pathologist’s annotation, the AI recognition is mostly correct. Green: true positive. Blue: true negative. Yellow: false positive (not tumor but recognized as tumor by AI). Red: false negative (tumor but recognized as not tumor by AI). **(D)** Representative fluorescence image with PD-L1 staining in green. **(E)** Nuclei detection by nuclei segmentation. In this image, it is not limited to the tumor area. **(F)** Result of PD-L1 membranous staining classification. Orange circle: PD-L1 positive. White circle: PD-L1 negative. (Scale bar: A-C: 500 μm, D-F: 100 μm)


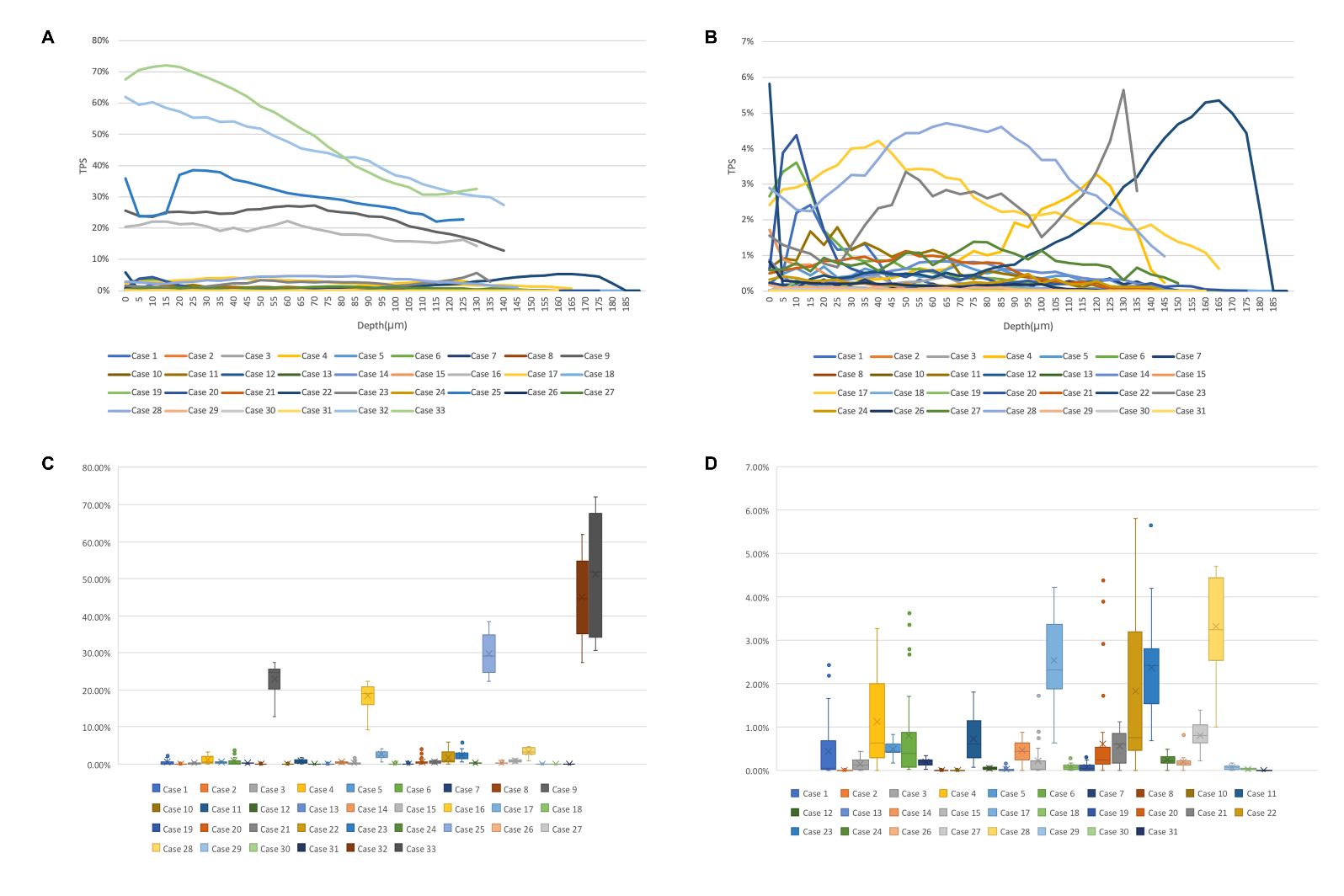


**Fig. S5. Relationship between PD-L1 TPS and tissue depth in all 33 pseudo-needle biopsy lung cancer specimens.**

**(A)** The PD-L1 TPS of each case across different tissue depth levels at 5 μm interval. Due to rehydrated section thickness variability, not every case has the same total depth analyzed. **(B)** The same data as shown in (A), excluding the five cases with TPS higher than 10%, highlighting the TPS variation in the low-expression cases. **(C)** Box plot of TPS of each case across the analyzed tissue depth, showing the median (-), average (x), interquartile range, maximal and minimal value. **(D)** The same data as shown in (C), excluding the five cases with TPS higher than 10%, highlighting the TPS variation in the low-expression cases.

**Table S1. Confusion matrix of tumor recognition model with cell instance accuracy calculation from testing dataset** (n=246470 cells)

|  | | **True condition** | |  |
| --- | --- | --- | --- | --- |
|  |  | Positive | Negative |  |
| **Predicted condition** | Positive | **True positive**  73883 | **False positive** 12651 |  |
|  | Negative | **False negative**  9602 | **True negative**  150334 |  |
|  | | **Sensitivity**  88.5% | **Specificity**  92.2% | **Accuracy**  91.0% |

**Table S2. Confusion matrix of PD-L1 membranous staining classification model with cell instance accuracy calculation from testing data set** (n=2544 cells)

|  | | **True condition** | |  |
| --- | --- | --- | --- | --- |
|  |  | Positive | Negative |  |
| **Predicted condition** | Positive | **True positive**  695 | **False positive** 122 |  |
|  | Negative | **False negative**  46 | **True negative**  1681 |  |
|  | | **Sensitivity**  93.8% | **Specificity**  93.2% | **Accuracy**  93.4% |

**Table S3. Concordance between AI-calculated PD-L1 TPS and pathologists’ evaluation**

1. Compared to pathologist YYL

|  | Pathologist’s TPS | | | |
| --- | --- | --- | --- | --- |
| AI TPS |  | <1% | 1-49% | ≥50% |
|  | <1% | 21 | 0 | 0 |
|  | 1-49% | 4 | 6 | 0 |
|  | ≥50% | 0 | 0 | 2 |

1. Compared to pathologist LCW

|  | Pathologist’s TPS | | | |
| --- | --- | --- | --- | --- |
| AI TPS |  | <1% | 1-49% | ≥50% |
|  | <1% | 19 | 2 | 0 |
|  | 1-49% | 6 | 4 | 0 |
|  | ≥50% | 0 | 2 | 0 |

1. Comparing TPS between the two pathologists

|  | Pathologist’s TPS (LCW) | | | |
| --- | --- | --- | --- | --- |
| Pathologist’s TPS (YYL) |  | <1% | 1-49% | ≥50% |
|  | <1% | 22 | 3 | 0 |
|  | 1-49% | 3 | 3 | 0 |
|  | ≥50% | 0 | 2 | 0 |

| **Table S4. Detailed clinical and pathological information of patients subjected to three-dimensional tumor PD-L1 expression characterization study** | | | | | | | | | |
| --- | --- | --- | --- | --- | --- | --- | --- | --- | --- |
| Case Number | Age at Time of Immunotherapy Start  or PD-L1 Testing | Sex | Smoking | Cancer Type | Diagnostic PD-L1 Antibody | PD-L1 Report | Therapeutic Anti-PD-1/PD-L1 Antibody | Best Clinical Response | Stage at Time of Immunotherapy Start  or PD-L1 Testing |
| 1 | 55 | F | No | Adenocarcinoma | 22C3 | TPS<1% (0%) | Atezolizumab | PD | rpM1a, stage IV |
| 2 | 54 | F | No | Adenocarcinoma | 22C3 | TPS<1% (0%) | Nivolumab | PD | rpM1b, stage IV |
| 3 | 39 | F | No | Adenocarcinoma, mucinous | 22C3 | TPS<1%(0%) | Nivolumab | PD | rpM1a, stage IV |
| 4 | 67 | F | No | Adenocarcinoma | 22C3 | TPS<1% (0%) | Pembrolizumab | PR | rpM1b, stage IV |
| 5 | 64 | F | No | Adenocarcinoma | 22C3 | TPS<1% (0%) | Atezolizumab | SD | rpM1b, stage IV |
| 6 | 55 | M | Yes | Pleomorphic carcinoma | 22C3 | TPS<1% (0%) | Atezolizumab | SD | rpM1b, stage IV |
| 7 | 58 | F | No | Adenosquamous carcinoma | 22C3 | TPS1-49%(5%) | None | NA | pT2aN0, stage IB |
| 8 | 64 | F | No | Adenocarcinoma | 22C3 | TPS1-49%(10%) | None | NA | rpTxN2Mx |
| 9 | 63 | M | Yes | Adenocarcinoma | 22C3 | TPS1-49%(5%) | Pembrolizumab | SD | pT3NxM1a stage IV |
| 10 | 83 | M | Yes | Adenocarcinoma | 22C3 | TPS<1%(0%) | Pembrolizumab | SD | rpM1b stage IV |
| 11 | 65 | F | No | Adenocarcinoma | 22C3 | TPS<1%(0%) | Atezolizumab | PD | rpM1b stage IV |
| 12 | 65 | F | No | Adenocarcinoma | 22C3 | TPS<1% (0%) | Nivolumab | SD | rpM1b, stage IV |
| 13 | 74 | M | No | Adenocarcinoma | 22C3 | TPS1-49%(5%) | Pembrolizumab | SD | rpM1b, stage IV |
| 14 | 74 | F | No | Adenocarcinoma | 22C3 | TPS<1% (0%) | Pembrolizumab | SD | rpM1a, stage IV |
| 15 | 43 | F | No | Adenocarcinoma | 22C3 | TPS1-49%(1%) | None | NA | pT2aN0M1a, stage IV |
| 16 | 41 | M | No | Adenocarcinoma | 22C3 | TPS1-49%(5%) | Atezolizumab | PD | rpM1b, stage IV |
| 17 | 74 | M | Yes | Adenocarcinoma | 22C3 | TPS1-49%(10%) | None | NA | rpM1b, stage IV |
| 18 | 70 | M | No | Combined large cell neuroendocrine carcinoma and adenocarcinoma | 22C3 | TPS1-49%(1%) | None | NA | rpM1a, stage IV |
| 19 | 64 | M | No | Adenocarcinoma | 22C3 | TPS1-49%(20%) | None | NA | rpM1a, stage IV |
| 20 | 63 | M | Yes | Adenocarcinoma | 22C3 | TPS1-49%(5%) | None | NA | pT2aN0M1a, stage IV |
| 21 | 80 | M | Yes | Adenocarcinoma | 22C3 | TPS>=50%(50%) | Nivolumab | PD | rpM1b stage IV |
| 22 | 70 | M | No | Adenocarcinoma | 22C3 | TPS1-49%(15%) | Pembrolizumab | Neoadjuvant and adjuvant setting, no relapse for 11 months | ypT2bN2Mx, stage IIIA |
| 23 | 63 | F | No | Adenosquamous carcinoma | 22C3 | TPS1-49%(5%) | Nivolumab | PR | rpM1b stage IV |
| 24 | 62 | M | Yes | Adenocarcinoma | SP142 | TC<1%(0%), IC>=10%(15%) | None | NA | rpT0N2M1a stage IV |
| 25 | 57 | M | Yes | Adenocarcinoma | SP263 | TPS >=50% (95%) | Nivolumab | SD | rpM1b, stage IV |
| 26 | 50 | F | No | Adenocarcinoma | 22C3 | TPS>=50%(80%) | Atezolizumab | PD | rpM1b, stage IV |
| 27 | 73 | M | No | Adenocarcinoma | 22C3 | TPS1-49%(15%) | None | NA | rpM1a, stage IV |
| 28 | 69 | M | Yes | Adenocarcinoma | 22C3 | TPS1-49%(40%) | Pembrolizumab | PR | rpM1b, stage IV |
| 29 | 65 | M | No | Pleomorphic carcinoma | 22C3 | TPS>=50%(60%) | Pembrolizumab | PD | rpM1b, stage IV |
| 30 | 65 | M | No | Adenocarcinoma | 22C3 | TPS>=50%(90%) | None | NA | pT3N2Mx, stage IIIa |
| 31 | 74 | F | No | Adenocarcinoma | SP263 | TPS <1% (<1%) | Atezolizumab | Adjuvant seetiing, no relapse for 4 months | pT2aN2M0, stage IIIA |
| 32 | 81 | F | No | Adenocarcinoma | 22C3 | TPS>=50%(90%) | None | NA | rpM1b stage IV |
| 33 | 57 | M | Yes | Adenocarcinoma | 22C3 | TPS>=50%(90%) | Pembrolizumab | Adjuvant setting, no relapse for 15 months | pT3N2M0 stage IIIB |
| TPS: tumor proportion score, NA: not applicable, SD: stable disease, PD: progressive disease, PR: partial response. The staging was according to American Joint Committee on Cancer 7th edition. | | | | | | | | | |
